# Supplementary material for: FACS-assisted single-cell lipidome analysis of phosphatidylcholines and sphingomyelins in cells of different lineages
Source: J Lipid Res. 2023 Feb 4;64(3):100341. doi: 10.1016/j.jlr.2023.100341 (PMC10027561; doi:10.1016/j.jlr.2023.100341)
Supplement: Supporting information [file mmc1.docx]

# Supporting Information for:

# FACS-assisted single cell lipidomics of phosphatidylcholines and sphingomyelins

Sarah E Hancock^1,2^*, Eileen Ding^1^, Emma Johansson Beves^3^, Todd Mitchell^4,5^, Nigel Turner^1,2^*

^1^Department of Pharmacology, School of Biomedical Sciences, UNSW Sydney, Australia.

^2^Cellular Bioenergetics Laboratory, Victor Chang Cardiac Research Institute, Sydney, NSW, Australia.

^3^Flow Cytometry Unit, Mark Wainwright Analytical Centre, UNSW Sydney, Australia

^4^School of Medicine, University of Wollongong, Wollongong Australia

^5^Molecular Horizons, University of Wollongong, Wollongong Australia

* corresponding author email [n.turner@victorchang.edu.au](mailto:n.turner@victorchang.edu.au) & [s.hancock@victochang.edu.au](mailto:s.hancock@victochang.edu.au).

## Tables

Table S1: Target list used for lipid identification in Lipidview software.

| ___Reference Peak Information | | | | | | | | |
| --- | --- | --- | --- | --- | --- | --- | --- | --- |
| **Name** | **Mass (m/z)** | **Precursor /NL of (Da)** | **Ion mode** | **Scan type** | **Class** | **Species** | **IS name** | **Isotopic correction factor** |
| IS PC 17:0/17:0 | 762.5 | 184.1 | Positive | PC |  |  | PC 34:0 | 1.642 |
| IS DHSM 12:0 | 649.5 | 184.1 | Positive | SM/PC |  |  | DHSM 12:0 | 1.516 |
| IS PE 17:0/17:0 | 720.5 | 141 | Positive | -PE |  |  | PE 34:0 | 1.588 |
| ___Target Lipid Information | | | | | | | | |
| PC 28:0 | 678.6 | 184.1 | Positive | SM/PC | PC | PC 28:0 | PC 34:0 | 1.535 |
| PC 30:1 | 704.6 | 184.1 | Positive | SM/PC | PC | PC 30:1 | PC 34:0 | 1.569 |
| PC 30:0 | 706.6 | 184.1 | Positive | SM/PC | PC | PC 30:0 | PC 34:0 | 1.57 |
| PC 32:2 | 730.6 | 184.1 | Positive | SM/PC | PC | PC 32:2 | PC 34:0 | 1.605 |
| PC 32:1 | 732.6 | 184.1 | Positive | SM/PC | PC | PC 32:1 | PC 34:0 | 1.605 |
| PC 32:0 | 734.6 | 184.1 | Positive | SM/PC | PC | PC 32:0 | PC 34:0 | 1.605 |
| PC 34:4 | 754.6 | 184.1 | Positive | SM/PC | PC | PC 34:4 | PC 34:0 | 1.641 |
| PC 34:3 | 756.6 | 184.1 | Positive | SM/PC | PC | PC 34:3 | PC 34:0 | 1.641 |
| PC 34:2 | 758.7 | 184.1 | Positive | SM/PC | PC | PC 34:2 | PC 34:0 | 1.641 |
| PC 34:1 | 760.6 | 184.1 | Positive | SM/PC | PC | PC 34:1 | PC 34:0 | 1.642 |
| PC 33:1 | 746.6 | 184.1 | Positive | SM/PC | PC | PC 33:1 | PC 34:0 | 1.64 |
| PC 33:0 | 744.7 | 184.1 | Positive | SM/PC | PC | PC 33:0 | PC 34:0 | 1.64 |
| PC O-36:5 | 766.7 | 184.1 | Positive | SM/PC | PC | PC O-36:5 | PC 34:0 | 1.674 |
| PC O-36:4 | 768.7 | 184.1 | Positive | SM/PC | PC | PC O-36:4 | PC 34:0 | 1.674 |
| PC O-36:3 | 770.6 | 184.1 | Positive | SM/PC | PC | PC O-36:3 | PC 34:0 | 1.675 |
| PC O-36:2 | 772.3 | 184.1 | Positive | SM/PC | PC | PC O-36:2 | PC 34:0 | 1.675 |
| PC O-36:1 | 774.4 | 184.1 | Positive | SM/PC | PC | PC O-36:1 | PC 34:0 | 1.675 |
| PC O-36:0 | 776.6 | 184.1 | Positive | SM/PC | PC | PC O-36:0 | PC 34:0 | 1.676 |
| PC 36:5 | 780.6 | 184.1 | Positive | SM/PC | PC | PC 36:5 | PC 34:0 | 1.678 |
| PC 36:4 | 782.6 | 184.1 | Positive | SM/PC | PC | PC 36:4 | PC 34:0 | 1.678 |
| PC 36:3 | 784.6 | 184.1 | Positive | SM/PC | PC | PC 36:3 | PC 34:0 | 1.678 |
| PC 36:2 | 786.6 | 184.1 | Positive | SM/PC | PC | PC 36:2 | PC 34:0 | 1.679 |
| PC 36:1 | 788.7 | 184.1 | Positive | SM/PC | PC | PC 36:1 | PC 34:0 | 1.679 |
| PC 36:0 | 790.3 | 184.1 | Positive | SM/PC | PC | PC 36:0 | PC 34:0 | 1.679 |
| PC O-38:6 | 792.4 | 184.1 | Positive | SM/PC | PC | PC O-38:6 | PC 34:0 | 1.712 |
| PC O-38:5 | 794.4 | 184.1 | Positive | SM/PC | PC | PC O-38:5 | PC 34:0 | 1.712 |
| PC O-38:4 | 796.4 | 184.1 | Positive | SM/PC | PC | PC O-38:4 | PC 34:0 | 1.712 |
| PC O-38:3 | 798.5 | 184.1 | Positive | SM/PC | PC | PC O-38:3 | PC 34:0 | 1.713 |
| PC O-38:2 | 800.8 | 184.1 | Positive | SM/PC | PC | PC O-38:2 | PC 34:0 | 1.713 |
| PC O-38:1 | 802.8 | 184.1 | Positive | SM/PC | PC | PC 38:8 | PC 34:0 | 1.715 |
| PC 38:7 | 804.6 | 184.1 | Positive | SM/PC | PC | PC 38:7 | PC 34:0 | 1.715 |
| PC 38:6 | 806.6 | 184.1 | Positive | SM/PC | PC | PC 38:6 | PC 34:0 | 1.715 |
| PC 38:5 | 808.6 | 184.1 | Positive | SM/PC | PC | PC 38:5 | PC 34:0 | 1.716 |
| PC 38:4 | 810.6 | 184.1 | Positive | SM/PC | PC | PC 38:4 | PC 34:0 | 1.716 |
| PC 38:3 | 812.7 | 184.1 | Positive | SM/PC | PC | PC 38:3 | PC 34:0 | 1.716 |
| PC 38:2 | 814.7 | 184.1 | Positive | SM/PC | PC | PC 38:2 | PC 34:0 | 1.717 |
| PC O-40:8 | 816.7 | 184.1 | Positive | SM/PC | PC | PC O-40:8 | PC 34:0 | 1.75 |
| PC O-40:7 | 818.7 | 184.1 | Positive | SM/PC | PC | PC O-40:7 | PC 34:0 | 1.75 |
| PC O-40:6 | 820.7 | 184.1 | Positive | SM/PC | PC | PC O-40:6 | PC 34:0 | 1.751 |
| PC O-40:5 | 822.7 | 184.1 | Positive | SM/PC | PC | PC O-40:5 | PC 34:0 | 1.751 |
| PC O-40:4 | 824.7 | 184.1 | Positive | SM/PC | PC | PC O-40:4 | PC 34:0 | 1.751 |
| PC O-40:3 | 826.4 | 184.1 | Positive | SM/PC | PC | PC O-40:3 | PC 34:0 | 1.752 |
| PC O-40:2 | 828.7 | 184.1 | Positive | SM/PC | PC | PC O-40:2 | PC 34:0 | 1.752 |
| PC 40:8 | 830.7 | 184.1 | Positive | SM/PC | PC | PC 40:8 | PC 34:0 | 1.752 |
| PC 40:7 | 832.5 | 184.1 | Positive | SM/PC | PC | PC 40:7 | PC 34:0 | 1.753 |
| PC 40:6 | 834.7 | 184.1 | Positive | SM/PC | PC | PC 40:6 | PC 34:0 | 1.755 |
| PC 40:5 | 836.7 | 184.1 | Positive | SM/PC | PC | PC 40:5 | PC 34:0 | 1.755 |
| PC 40:4 | 838.5 | 184.1 | Positive | SM/PC | PC | PC 40:4 | PC 34:0 | 1.755 |
| PC 40:3 | 840.6 | 184.1 | Positive | SM/PC | PC | PC 40:3 | PC 34:0 | 1.756 |
| PC 40:2 | 842.5 | 184.1 | Positive | SM/PC | PC | PC 40:2 | PC 34:0 | 1.756 |
| SM 32:1;2 | 675.6 | 184.1 | Positive | SM/PC | SM | SM 32:1;2 | DHSM 12:0 | 1.55 |
| SM 34:2;2 | 701.6 | 184.1 | Positive | SM/PC | SM | SM 34:2;2 | DHSM 12:0 | 1.586 |
| SM 34:1;2 | 703.6 | 184.1 | Positive | SM/PC | SM | SM 34:1;2 | DHSM 12:0 | 1.586 |
| SM 34:0;2 | 705.6 | 184.1 | Positive | SM/PC | SM | SM 34:0;2 | DHSM 12:0 | 1.586 |
| SM 42:2;2 | 813.6 | 184.1 | Positive | SM/PC | SM | SM 42:2;2 | DHSM 12:0 | 1.736 |
| SM 42:1;2 | 815.6 | 184.1 | Positive | SM/PC | SM | SM 42:1;2 | DHSM 12:0 | 1.736 |
| PE 32:2 | 688.7 | 141 | Positive | -PE | PE | PE 32:2 | PE 34:0 | 1.551 |
| PE 32:1 | 690.7 | 141 | Positive | -PE | PE | PE 32:1 | PE 34:0 | 1.552 |
| PE 32:0 | 692.7 | 141 | Positive | -PE | PE | PE 32:0 | PE 34:0 | 1.552 |
| PE 34:3 | 714.6 | 141 | Positive | -PE | PE | PE 34:3 | PE 34:0 | 1.586 |
| PE 34:2 | 716.6 | 141 | Positive | -PE | PE | PE 34:2 | PE 34:0 | 1.587 |
| PE 34:1 | 718.6 | 141 | Positive | -PE | PE | PE 34:1 | PE 34:0 | 1.587 |
| PE O-36:5 | 724.6 | 141 | Positive | -PE | PE | PE O-36:5 | PE 34:0 | 1.6183 |
| PE 36:6 | 736.6 | 141 | Positive | -PE | PE | PE 36:6 | PE 34:0 | 1.622 |
| PE 36:5 | 738.6 | 141 | Positive | -PE | PE | PE 36:5 | PE 34:0 | 1.622 |
| PE 36:4 | 740.7 | 141 | Positive | -PE | PE | PE 36:4 | PE 34:0 | 1.622 |
| PE 36:3 | 742.6 | 141 | Positive | -PE | PE | PE 36:3 | PE 34:0 | 1.623 |
| PE 36:2 | 744.6 | 141 | Positive | -PE | PE | PE 36:2 | PE 34:0 | 1.623 |
| PE 36:1 | 746.7 | 141 | Positive | -PE | PE | PE 36:1 | PE 34:0 | 1.623 |
| PE 36:0 | 748.6 | 141 | Positive | -PE | PE | PE 36:0 | PE 34:0 | 1.624 |
| PE O-38:6 | 750.6 | 141 | Positive | -PE | PE | PE O-38:6 | PE 34:0 | 1.6549 |
| PE O-38:2 | 758.8 | 141 | Positive | -PE | PE | PE O-38:2 | PE 34:0 | 1.6562 |
| PE 38:7 | 762.5 | 141 | Positive | -PE | PE | PE 38:7 | PE 34:0 | 1.658 |
| PE 38:6 | 764.6 | 141 | Positive | -PE | PE | PE 38:6 | PE 34:0 | 1.658 |
| PE 38:5 | 766.6 | 141 | Positive | -PE | PE | PE 38:5 | PE 34:0 | 1.659 |
| PE 38:4 | 768.6 | 141 | Positive | -PE | PE | PE 38:4 | PE 34:0 | 1.659 |
| PE 38:3 | 770.6 | 141 | Positive | -PE | PE | PE 38:3 | PE 34:0 | 1.659 |
| PE 38:2 | 772.7 | 141 | Positive | -PE | PE | PE 38:2 | PE 34:0 | 1.66 |
| PE O-40:2 | 786.6 | 141 | Positive | -PE | PE | PE O-40:2 | PE 34:0 | 1.6939 |
| PE 40:8 | 788.6 | 141 | Positive | -PE | PE | PE 40:8 | PE 34:0 | 1.696 |
| PE 40:7 | 790.6 | 141 | Positive | -PE | PE | PE 40:7 | PE 34:0 | 1.696 |
| PE 40:6 | 792.6 | 141 | Positive | -PE | PE | PE 40:6 | PE 34:0 | 1.696 |
| PE 40:5 | 794.6 | 141 | Positive | -PE | PE | PE 40:5 | PE 34:0 | 1.697 |
| PE 40:2 | 800.8 | 141 | Positive | -PE | PE | PE 40:2 | PE 34:0 | 1.698 |
| PE O-42:4 | 810.5 | 141 | Positive | -PE | PE | PE 41:4 | PE 34:0 | 1.7318 |
| PE O-42:2 | 814.6 | 141 | Positive | -PE | PE | PE 41:2 | PE 34:0 | 1.7325 |

*DHSM* dihydrosphingomyelin, *IS* internal standard, *NL* neutral loss, *PC* phosphatidylcholine, *PE* phosphatidylethanolamine

Table S2: Statistical output for all detected PC and SM species significantly different between bulk cell extracts and fifty cells obtained by FACS for both C2C12 and HepG2 cells grown in control or DHA-supplemented media

|  |  |  | Mean values* | | |  |  |  |  |  |
| --- | --- | --- | --- | --- | --- | --- | --- | --- | --- | --- |
| Cell line | **Lipid species** | **Media** | **Bulk cell extract** | **Fifty cells** | **Difference** | **statistic** | **p.value** | **parameter** | **conf.low** | **conf.high** |
| C2C12 | PC 28:0 | CON | 0.677267 | 0.057315 | 0.619952 | 7.711064 | 0.007596 | 2.576296 | 0.338557 | 0.901348 |
| C2C12 | PC 40:5 | DHA | 1.136988 | 0.470475 | 0.666513 | 4.506505 | 0.027431 | 2.604082 | 0.152637 | 1.180389 |
| C2C12 | PC O-38:3 | CON | 1.503391 | 0.319038 | 1.184353 | 4.514423 | 0.018057 | 3.16842 | 0.373996 | 1.99471 |
| C2C12 | PC O-38:4 | DHA | 2.905955 | 1.597656 | 1.308299 | 3.842199 | 0.029799 | 3.07324 | 0.239115 | 2.377482 |
| C2C12 | PC O-40:3 | DHA | 0.45247 | 1.892797 | -1.44033 | -3.80111 | 0.02208 | 3.692901 | -2.5278 | -0.35285 |
| C2C12 | PC O-40:5 | DHA | 1.854475 | 1.109687 | 0.744788 | 3.188368 | 0.042093 | 3.383427 | 0.046844 | 1.442733 |
| C2C12 | SM 34:0 | CON | 1.533367 | 0.051599 | 1.481768 | 5.949405 | 0.022071 | 2.17902 | 0.490243 | 2.473292 |
| C2C12 | SM 34:0 | DHA | 0.948997 | 0.218288 | 0.730709 | 3.42506 | 0.039521 | 3.106405 | 0.064729 | 1.39669 |
| C2C12 | SM 34:2 | CON | 1.819516 | 0 | 1.819516 | 22.48913 | 0.001971 | 2 | 1.471403 | 2.167628 |
| C2C12 | SM 34:2 | DHA | 2.868861 | 0.659312 | 2.20955 | 5.528401 | 0.026605 | 2.146535 | 0.597638 | 3.821462 |
| HepG2 | PC 33:0 | CON | 0 | 1.173872 | -1.17387 | -11.9612 | 0.006917 | 2 | -1.59613 | -0.75161 |
| HepG2 | PC O-36:0 | CON | 0 | 0.263927 | -0.26393 | -8.45971 | 0.013687 | 2 | -0.39816 | -0.12969 |
| HepG2 | PC O-36:5 | DHA | 0.037858 | 0.307606 | -0.26975 | -9.96044 | 6.67E-04 | 3.875572 | -0.3459 | -0.19359 |
| HepG2 | PC O-38:4 | CON | 0.908009 | 0.209331 | 0.698678 | 3.284398 | 0.042226 | 3.195523 | 0.044529 | 1.352827 |
| HepG2 | PC O-40:2 | CON | 0.011522 | 0.583806 | -0.57228 | -7.47863 | 0.015332 | 2.092745 | -0.88794 | -0.25663 |
| HepG2 | PC O-40:3 | CON | 0.181543 | 1.176038 | -0.9945 | -5.11354 | 0.012925 | 3.140889 | -1.59801 | -0.39098 |
| HepG2 | PC O-40:5 | CON | 0.005649 | 0.280797 | -0.27515 | -19.382 | 5.31E-04 | 2.726945 | -0.323 | -0.2273 |
| HepG2 | PC O-40:5 | DHA | 9.94E-04 | 0.256989 | -0.256 | -6.05471 | 0.026142 | 2.002211 | -0.43772 | -0.07427 |
| HepG2 | SM 32:1 | CON | 1.193725 | 0.286911 | 0.906814 | 3.49325 | 0.041589 | 2.910934 | 0.066196 | 1.747433 |
| HepG2 | SM 34:2 | CON | 0.652823 | 0 | 0.652823 | 11.34558 | 0.007679 | 2 | 0.405249 | 0.900397 |

*Mean values calculated from the relative abundance of PC & SM species (n=3). Welch t test, *P* < 0.05

## Figures


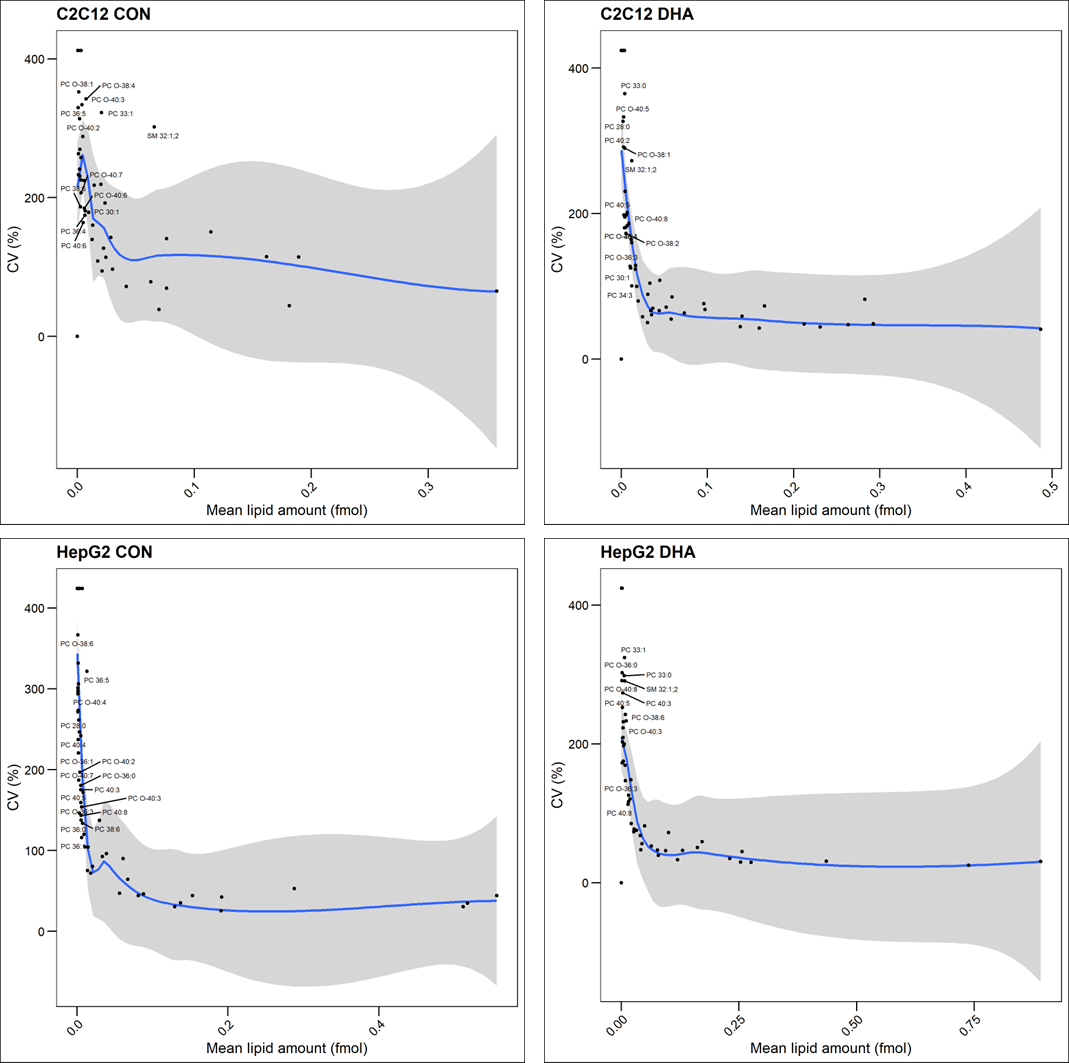


Figure S1: Association between the coefficient of variation (CV) and mean amount of lipid (fmol) measured in single C2C12 and HepG2 cells grown in control media (CON) or supplemented with 50 uM docosahexaenoic acid (DHA) overnight. Regression line (blue) was calculated by local polynomial regression fitting (loess) with grey shaded area showing the 95% confidence interval. Lipid species that fall outside of the 95% confidence interval are labelled.


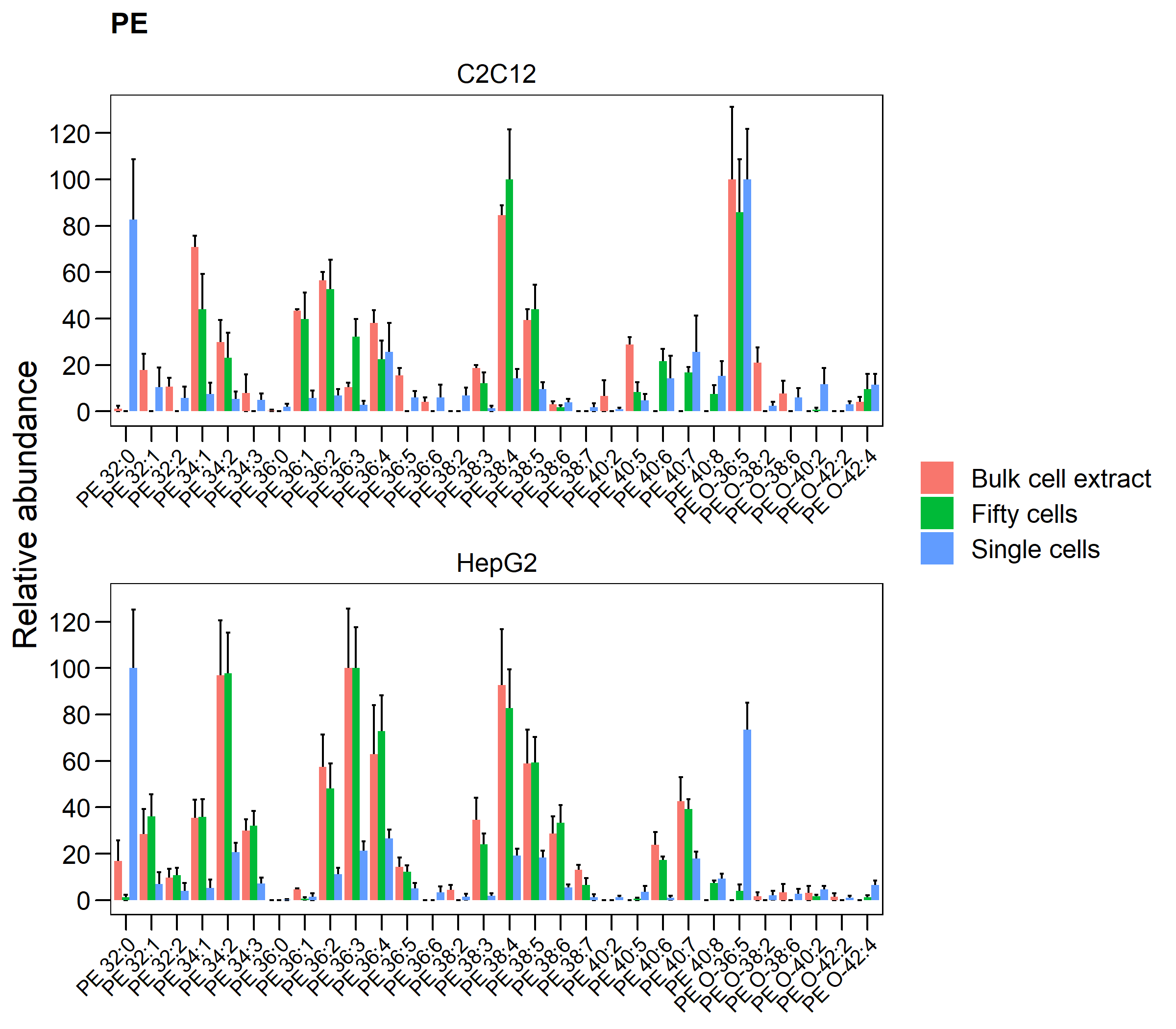


Figure S2: relative abundance of phosphatidylethanolamine (PE) species detected from bulk cell extracts (n=3), fifty sorted cells (n=3) and single sorted cells (n=17-18) by shotgun lipidomics. Values are mean(±SEM).
